# Leveraging risk communication and community engagement and lessons from previous outbreaks to strengthen a Public Health response: A case study of Disease X in the Panzi region, DRC

**DOI:** 10.4102/jphia.v16i1.1322

**Published:** 2025-06-30

**Authors:** Pierre Gashema, Patrick G. Iradukunda, Placide Sesonga, Radjabu Bigirimana, Jean C. Mugisha, Jean dD. Harelimana, Mosoka P. Fallah, Tafadzwa Dzinamarira, Claude M. Muvunyi

**Affiliations:** 1 Africa Centres for Disease Control and Prevention, Addis Ababa, Ethiopia; 2 Research Department, Repolicy Research Centre, Kigali, Rwanda; 3 Drugs Department, Rwanda Food and Drugs, Authority, Kigali, Rwanda; 4 Division of Clinical Medicine, University of Global Health Equity, Kigali, Rwanda; 5 College of Medicine and Health Sciences, University of Rwanda, Kgali, Rwanda; 6 World Health Organization, Kigali, Rwanda; 7 School of Health Systems and Public Health, University of Pretoria, Pretoria, South Africa; 8 Rwanda Biomedical Centre, Kigali, Rwanda

**Keywords:** Disease X, outbreak response, risk communication, surveillance systems, Kwango province, DRC

## Abstract

On 08 December 2024, the World Health Organization (WHO) reported an outbreak of Disease X in the Panzi Health Zone, Kwango province, Democratic Republic of the Congo (DRC). This unknown pathogen, with 406 cases and 31 deaths at the time of its declaration, predominantly affects children under 5 years. Disease X, hypothesised to be a zoonotic ribonucleic acid (RNA) virus, poses significant challenges because of limited healthcare infrastructure, gaps in risk communication and ineffective community engagement. This opinion article aims to explore these challenges and advocate for the urgent need for culturally tailored, inclusive communication strategies that foster trust and empower local communities in responding to outbreaks. Key approaches highlighted include mobilising local leaders, utilising mobile laboratories for decentralised diagnostics and improving sample collection techniques. Drawing on lessons from previous epidemics, such as COVID-19 and Ebola, this article emphasises the importance of robust surveillance systems, community engagement and effective risk communication, skilled health workforce and collaborative management frameworks. Strengthening early warning systems and ensuring equitable access to diagnostic and treatment resources are essential for mitigating future outbreaks of unknown diseases in resource-limited settings.

## Introduction

On 08 December 2024, the World Health Organization (WHO) issued a ‘Situation at a Glance’, announcing that the Democratic Republic of the Congo (DRC) had reported an undiagnosed illness, referred to as Disease X, caused by an unknown pathogen.^1^ As of 16 December 2024, 406 cases and 31 deaths were reported in the Panzi health zone, located in Kwango province, one of the remote areas of DRC. The Disease X mainly affects children under 5 years.^2^ Disease X refers to an unknown pathogen with the potential to cause a global epidemic or pandemic. Currently, WHO has classified Disease X among the high priorities for research and development.^3^ Disease X is hypothesised to result from a ‘pathogen X’, most likely an ribonucleic acid (RNA) virus, suspected to be transmitted by zoonotic agents.^3^ On 06 December 2024, the Africa Centers for Disease Control and Prevention (CDC) led the response and deployed experts for investigating the causative agents of Disease X.^4^ The key challenges during the response to Disease X included uncertainty about transmission routes, which hampered effective prevention measures, and gaps in communication and community engagement.^4^ The Panzi health zone is located in a remote region, over 48 h and 700 km by road from Kinshasa. This region faces challenges, including limited laboratory capacity, geographic isolation and resource constraints, and these hindrances may delay outbreak response efforts.^4^ The recent report highlighted that the isolation of the Panzi region hampered the collection of samples timely.^4^ In addition, escalating food insecurity in the same region may have contributed to the propagation of disease, but data are scarce. Moreover, inadequate local systems for disease surveillance, shortage of human resources and infrastructures further accentuate the outbreak.^2^ Political instability and shortage of funds in the health sector also left the population vulnerable in the Panzi region.^2,4^ Risk communication and community engagement (RCCE) are critical during any disease outbreak, especially in remote areas where there is limited healthcare infrastructure and a vulnerability to infectious disease outbreaks.^5^ Effective RCCE ensures that communities understand the nature of the outbreak, enabling them to take preventive actions such as isolating suspected cases, seeking early treatment and adhering to public health guidelines.^6^ Community engagement is equally vital, as it empowers local leaders, religious groups and community health workers to play active roles in outbreak response, fostering cooperation and accountability.^7^ During the COVID-19 pandemic, engaging community leaders helped to mobilise communities for effective response, and similarly, in the context of Disease X, involving communities in designing and implementing response strategies ensures that interventions are culturally acceptable and contextually relevant, improving adherence to control measures. Given the situation in the Panzi health zone of the DRC, there is an urgent need to strengthen RCCE measures to enhance the response to the current outbreak of Disease X. The lessons drawn from the previous deadly outbreaks can be instrumental to respond to Disease X. This opinion article aims to explore the best approaches for RCCE and other strategies to enhance the response to Disease X in the Panzi region of the DRC.

## Health communication tools for the prevention and control of Disease X in the Panzi region

Effective communication strategies have been proven to be instrumental for the control of outbreak.^6^ To tackle Disease X outbreak in the Panzi region, trust, accessibility and community involvement should be prioritised. The Ministry of Health should capacitate the community health workers’ (CHWs) workforce to clearly transmit the messages in local languages, preferably in a door-to-door fashion. Radio broadcasts and mobile loudspeakers can also be used in hard-to-reach areas. Key messages should focus on the awareness of the symptoms of disease, infection and prevention strategies (IPC). Social influencers and religious leaders are also the key population to communicate the messages related to outbreaks. Moreover, to reinforce the awareness, visual aids providing the details on Disease X can be posted in all public spaces.

Feedback loops through surveys and focus groups can help to refine approaches, addressing fear and stigma. These strategies foster community trust and cooperation, ensuring a robust response and preparedness for future health crises. During the COVID-19 outbreak, different approaches were employed to interrupt disease transmission such as lockdowns; curfews; establishing the washing hands stations in public and private business facilities, such as marketplaces, churches, hospitals, and schools; and social distancing.^8^ Effective IPC and community engagement approaches were used to timely control Ebola and Marburg diseases in the Western and Eastern African regions. Tailored approaches based on these lessons learnt can be employed to control disease under investigation such as Disease X in the Panzi region, DRC.

## Leveraging mobile handsets for effective health communication

In limited-resource settings and hard-to-reach areas, mobile handsets serve as vital tools for real-time health communication, bridging gaps in healthcare access.^9^ During Disease X outbreaks in fragile states, they enable rapid dissemination of lifesaving information, support surveillance and facilitate telemedicine for frontline workers. Mobile messaging platforms enhance RCCE by providing timely updates, combating misinformation and ensuring community trust.^5^ In addition, short message service (SMS) alerts and mobile health apps improve adherence to public health measures and vaccination campaigns. Leveraging mobile technology during Disease X strengthens outbreak preparedness, enhances response coordination and ultimately mitigates the impact of emerging health threats.

## Strengthening community engagement through interactive health communication

To enhance communication for Disease X in rural areas, it is imperative to establish feedback mechanisms for integrating community-based approaches of using traditional leaders, community leaders, religious group leaders, school-based programmes and CHWs to allow feedback between health systems and communities. In addition, hotlines, suggestion boxes and social media platforms can be used as tools for collecting community concerns. Furthermore, interactive sessions such as Question and Answer (Q&A) events, workshops and community dialogues aim to address misconceptions and provide accurate information. These efforts build trust, ensure community engagement through interactive health communication and empower communities to participate in disease prevention, fostering timely reporting and adherence to public health interventions. These communication mechanisms have been used in various countries for outbreak response and disease surveillance. In Rwanda, the Ministry of Health established hotlines and suggestion boxes in all health facilities to facilitate the patients to timely share all information related to COVID-19, Rift valley, mpox and Marburg disease. In addition, Rwanda Health Communication Center initiated the use of social media hashtags for receiving community concerns and dissemination of information.^10^ Rwanda also used citizens’ assemblies through local government for Q&A sessions for enhancing community participation. This strategy played vital roles for awareness during the COVID-19 pandemic. Democratic Republic of Congo’s response to Disease X may also leverage social media and integrated community-based approaches for dynamic and inclusive communication.

## Focus on inclusivity

An equity-based approach for Disease X in remote areas involves tailoring health messages for marginalised and at-risk groups, including refugees and indigenous populations. Emphasising gender sensitivity, prioritising the roles of women as caregivers, equipping them with targeted education on prevention and treatment for under-fives are essential for inclusion of the most affected group. Public health leaders should establish plans to empower women through inclusive engagement that fosters community resilience, ensuring that interventions address both gender-specific needs and the broader vulnerabilities of marginalised populations.

## Recommendations on contextual challenges and response to Disease X

The outbreak of Disease X presents numerous challenges, particularly in regions with limited healthcare infrastructure and resources. To effectively limit the transmission of disease, it is essential to address both the contextual challenges and the response strategies necessary for robust, coordinated efforts. Key considerations include strengthening early detection through mobile laboratories, improving supply chain management for essential health resources and enhancing workforce capacity, especially in remote areas. In addition, the use of innovative technologies, such as drones, equipped with artificial intelligence (AI) technology for sample transportation and delivering essential supplies, can play pivotal roles in remote areas.

## Availability of mobile laboratories

Early detection is the cornerstone for a proper response to the outbreak. Africa, as a continent experiencing the outbreak, should employ the mobile laboratory in the hotspot areas for bridging the patient’s journey and closing the chain of transmission. Lessons learnt from the COVID-19 and Ebola outbreaks have reinforced the use of mobile laboratories. These approaches have been used in East African communities to strengthen outbreak response and surveillance.^11^ To effectively respond to Disease X in remote areas, mobile laboratories can play instrumental roles. In addition, public health leaders should consider decentralising cost-effective and portable diagnostic tools such as lateral flow assay and isothermal amplification.

### Calling to use deoxyribonucleic acid or ribonucleic acid shield for sample collection for preserving integrity of pathogens

Using A deoxyribonucleic acid (DNA) or ribonucleic acid (RNA) shield in sample collection is crucial in resource-limited settings and for long distances to national laboratories. It stabilises nucleic acids, preserving sample integrity without requiring cold-chain transport.^12^ This ensures accurate molecular diagnostics, critical for outbreak response, epidemiological surveillance and disease control, especially where timely access to laboratory facilities is challenging, reducing degradation and contamination risks.

### Robust supply chain management

Developing strategies to ensure continuous availability of essential supplies, such as diagnostic kits, personal protective equipment (PPE) and treatment options, leveraging partnerships with regional and international stakeholders. The Africa CDC pooled procurement mechanism has proven effective in responding to outbreaks such as Ebola, mpox, Marburg and COVID-19. In a strategic partnership, the Pan American Health Organization (PAHO) and the Africa CDC aim to enhance equitable access to vaccines, medicines and health technologies by strengthening regulatory frameworks, fostering innovation and bolstering production.^13^ This collaboration leverages PAHO’s Regional Revolving Funds and supports Africa’s initiatives in pooled procurement and local manufacturing to benefit both regions.

### Strengthening health workforce capacity and collaboration

Based on lessons learned from previous outbreaks, it is crucial to strengthen the capacity of the health workforce at all levels, from CHWs to national healthcare systems. Marginalised populations often have their primary interactions with CHWs, making them vital for effective community-level response and prevention efforts. Enhancing the skills, resources and support available to CHWs will play a pivotal role in bolstering community resilience and improving outbreak management.^14^ Moreover, it will also help in comprehensive preparedness strategies, including vigilant surveillance and resilient health systems. Skilled workforce development and effective training programmes can significantly enhance healthcare workers’ knowledge, confidence and safety.

### Use of drones for delivering essential medicine and transportation of critical samples

The unique geographical and logistical challenges across Africa necessitate the use of innovative technologies to strengthen supply chain systems, particularly in remote and underserved areas. Drones have emerged as a critical solution for addressing delays in the delivery of essential medicines, vaccines, food supplements and blood components, thereby significantly reducing patient waiting times and improving health outcomes. Countries such as Rwanda, Ghana, Uganda, Kenya, Guinea-Conakry and Nigeria have successfully integrated drone technology into their healthcare systems, demonstrating its effectiveness in bridging access gaps.^15^ Similarly, in resource-limited settings such as the Panzi region, employing drones during a Disease X outbreak could ensure the timely transport of critical samples to central laboratories, facilitating rapid diagnosis and informed public health interventions. Integrating these technologies into emergency response frameworks will enhance resilience and preparedness for future health crises.

### Collaborative management of Disease X in the Democratic Republic of Congo

Effective management of Disease X in the DRC requires strong collaboration between governmental agencies, non-governmental organisations (NGOs) and international partners. Establishing Inter-Agency Coordinating Committees (ICCs) can enhance strategic alignment, optimise resource allocation and streamline outbreak response efforts. A well-coordinated, multi-sectoral approach will facilitate rapid information sharing, strengthen surveillance systems and improve access to diagnostics, treatment and preventive measures, ultimately enhancing the country’s resilience to emerging infectious threats.

## Conclusion

The emerging outbreaks are not predictable, and this underscores the urgent need for a proactive, evidence-based public health approach. Strengthening early warning systems through digital health platforms and community-based surveillance will be critical in detecting emerging threats. Investments in resilient healthcare infrastructure must prioritise scalable diagnostic, treatment and isolation capacities, alongside robust supply chains for essential medical resources. Effective risk communication strategies that foster public trust and community engagement will enhance response efforts. Advancing research on rapid diagnostics, vaccines and therapeutics through global collaboration will ensure timely and equitable access during health emergencies.
